# Influence of game-based learning in mathematics education on the students' cognitive and affective domain: A systematic review

**DOI:** 10.3389/fpsyg.2023.1105806

**Published:** 2023-03-28

**Authors:** Hii Bii Hui, Muhammad Sofwan Mahmud

**Affiliations:** Faculty of Education, Universiti Kebangsaan Malaysia, Bangi, Selangor, Malaysia

**Keywords:** affective domain, cognitive domain, game-based learning, mathematics education, teaching and learning

## Abstract

**Introduction:**

Game-based learning (GBL) is one of the modern trends in education in the 21st century. Numerous research studies have been carried out to investigate the influence of teaching on the students' academic attainment. It is crucial to integrate the cognitive and affective domains into teaching and learning strategies. This study aims to review journal articles from 2018 to 2022 concerning the influence of GBL in mathematics T&L on the students' cognitive and affective domains.

**Methods:**

A research methodology based on PRISMA (Preferred Reporting Items for Systematic Reviews and Meta-Analyses) was used for the survey on the basis of the Scopus and Web of Science (WOS) databases wherein 773 articles relating to game-based learning (GBL) in mathematics were discovered. Based on the study topic, study design, study technique, and analysis, only 28 open-access articles were chosen for further evaluation. Two types of cognitive domain and five types of affective domain were identified as related to the implications of GBL on the students' T&L of mathematics.

**Results:**

The study results show that GBL has positively impacted students when they are learning mathematics. It is comprised of two types of cognitive domain (knowledge and mathematical skills) and five types of affective domain (achievement, attitude, motivation, interest, and engagement). The findings of this study are anticipated to encourage educators in the classrooms more effectively.

**Discussion:**

GBL in education is now one of the major learning trends of the 21st century. Since 2019, the number of studies relating to game-based learning has increased. There is an influence on the cognitive and affective domains due to T&L Mathematics utilizing a game-based learning (GBL) approach.

## 1. Introduction

Educational transformation is necessary because the success of said economic transformation is very much dependent on the successfulness of a futuristic education plan (Leal Filho et al., 2018). Globalization has formed a new path in worldwide education, and teachers play an active role in the teaching and learning process. In this regard, pedagogy is stressing more about the roles of the students in the learning sessions, specifically how it is compatible with the 21st century learning methods (Amran et al., 2019). According to Kamarudin et al. (2019), the level of student interest in teaching and learning is low when the conventional approach is employed. Consequently, teaching methods and techniques are essential for becoming a teacher who can impart knowledge to their pupils using a variety of engaging approaches and strategies. Teachers must employ the most effective method for imparting knowledge.

Subsequently, learning is intimately tied to the learning domains (Bloom et al., 1956) and has been introduced to education to encourage higher order thinking (Bitok, 2020). It encompasses the cognitive, affective, and psychomotor domains specifically. The cognitive domain is concerned with the intellectual growth of students, the affective domain involves the development of the students' attitudes, feelings, and values, while the psychomotor domain involves the physical development of the students. In this scenario, the cognitive and emotive domains of the pupils also influence the effectiveness of the GBL method.

GBL in education is now one of the major learning trends of the 21st century (Ahmad and Iksan, 2021) and it has received an increasing amount of academic attention in recent years (Zou, 2020). GBL is a mathematics teaching technique that creates a balance between classroom learning and educational games while enhancing the learning efficiency through student-centered learning activities (Lasut and Bawengan, 2020). It is also one of the more creative and entertaining methods, and, indirectly, students will pay attention to the teacher's lessons. This is due to the fact that playing games is innate to the students. Additionally, educational games may encourage the students to enjoy learning, to feel comfortable approaching a variety of difficulties along the way, and to overcome these challenges with focus, self-assurance, and patience, all of which are crucial for higher education in the development of lifelong learners (Liu et al., 2021).

This strategy is also founded in constructivist learning which emphasizes the importance of experiential learning through social interactions with the environment and their peers (Hourdequin et al., 2017). There is substantial data indicating that GBL is becoming increasingly popular as an effective learning approach utilized to create an engaging learning environment. On the basis of the empirical evidence from recent studies, the effectiveness of digital games in the education context has further proven the potential of GBL in boosting motivation, engagement, and social influences (Hernández-lara and Serradell-lopez, 2018).

According to Wong and Osman (2018), there are two types of game: digital and non-digital games. GBL, in the form of digital or non-digital games, aims to achieve the learning objectives set. According to Khairuddin and Mailok (2019), the GBL approach is used to stimulate and motivate the students to participate more actively in the learning process, to make the learning process more enjoyable, and to assist the students in comprehending the lessons more effectively. The GBL technique enables teachers to include active learning in their lessons, to increase the students' interest and engagement, and to receive instant feedback from the students' performance.

It should be noted that teachers should pay close attention to how gamification affects their student's interactions, emotionality, and cognitive activity—three aspects of the educational process. However, the acceptance and engagement of gamification in pedagogy remains challenging (Ding et al., 2018). The implementation of the gamification techniques is less appropriate to be carried out when the pupils have special needs (Mohamed Rosly and Khalid, 2017). This is due to the fact that the competence level of the pupil will affect the effectiveness of the implementation of gamification. In a general sense, this systematic literature review (SLR) was conducted to identify the influence on the cognitive and affective domains due to T&L Mathematics utilizing a game-based learning (GBL) approach.

### 1.1. The review protocol—PRISMA

This review was guided by PRISMA, developed by Page et al. (2021) with the aim of complete reporting to allow readers to assess the appropriateness of the methods used. In addition, presenting and summarizing the characteristics of the studies contributing to a synthesis allows policymakers to evaluate the applicability of the findings to their settings. A systematic review was chosen to describe, evaluate and synthesize the current empirical studies on the implications on the cognitive and affective domains of pupils due to game-based learning (GBL) methods in the teaching and learning of mathematics (T&L). Consideration was given to the Preferred Reporting Items for Systematic Reviews and Meta-Analyses (PRISMA) statement as a guideline to ensure that the research was conducted systematically (Moher et al., 2009).

### 1.2. Systematic search strategy

To find the relevant papers, there were four systematic techniques (identification, screening, eligibility, and included) used in this phase. The authors were able to completely discover and synthesize the research using these techniques, resulting in a well-organized and transparent systematic literature review.

Two databases, Scopus and Web of Science (WOS), were utilized for searching for previous research articles. The Scopus database is a library database that indexes the abstracts and citations of the scientific journal articles owned by Elsevier, a major journal publisher in the world offering high-impact papers. The Scopus database can be accessed through the off-campus access service for students provided by Tun Seri Lanang Library in the National University Malaysia *via* the website https://login.ezplib.ukm.my/menu. The Web of Science (WOS) database was chosen because it is an online digital library for research and educational information and a repository that specializes in the education field. Setting the appropriate keywords at this stage was essential for generating articles that met the study objectives.

#### 1.2.1. Phase 1: Identification

“Influence of Game-Based Learning in Mathematics Education on the Students' Cognitive and Affective Domains” is the title of this study. This study's key contribution is its evaluation of the impact of game-based mathematics instructions on student cognitive and affective functioning. As a result, data from the Scopus and Web of Science (WOS) databases was used in this study, along with the keywords game or game-based, mathematics or math, and affective or domain. The researcher read the article titles and abstracts after conducting a keyword search to identify articles relevant to game-based learning in mathematical education. The identification phase revealed 353 articles from Scopus and 420 articles from the Web of Science (WOS) using the search items “gamification,” “affective domain,” and “mathematics” along with Open Access and fixed operators. The number of detected articles was 773 in total. Some duplicate articles across the two databases were detected and eliminated. Table 1 below shows the specific keywords used for the database.

**Table 1:** Specific keywords used for the databases.

**Database**	**Website**	**Search key words**
Scopus	https://www.scopus.com/home.uri	TITLE-ABS-KEY ((“game” OR “game-based”) AND (“mathematics” OR “math”) AND (“affective*” OR “domain*” OR “influence*”))
Web of Science (WOS)	https://www.webofscience.com/wos/woscc/basic-search	TS = ((“game” OR “game-based”) AND (“mathematics” OR “math”) AND (“affective*” OR “domain*” OR “influence*”))

#### 1.2.2. Phase 2: Screening

The screening process occurred after identifying the articles. The articles were then either included or excluded from the study based on a specific set of criteria. The listed criteria were determined by the researchers (Mohamed Shaffril et al., 2021). The criteria considered for this study were adapted from several other SLR studies, namely the studies by Margot and Kettler (2019), Mohamed Shaffril et al. (2020), Mat and Mohd Matore (2020), and Amalina et al. (2021). The criteria that have been set were (i) articles published from 2018 to 2022, (ii) articles from journals only, (iii) articles related to the mathematics learning research field at the school level only, (iv) full-text articles, and (v) articles that have empirical data (Mat and Mohd Matore, 2020). It was carefully adapted to match the study requirements. The inclusion and exclusion criteria for this study are listed in Table 2.

**Table 2:** Article inclusion and exclusion criteria set.

**Process**	**Selection limits**	**Included**	**Excluded**
Criteria	Year	Articles starting 2018	Articles before 2018
	Type of publishing	Journal Articles	*Books, Proceedings of Conferences, Book Subtitles, Theses, Newspaper Clippings*
	Field of study	Mathematics learning	Apart from mathematics learning
	Access	Full access articles	Articles with limited access
	Type of research	Empirical data research	Non-empirical data research

Articles published prior to 2016, chapters from books and journals, review pieces, publications from proceedings, and articles written in languages other than English and Malay were among the 522 articles eliminated at this stage. The screening revealed a total of 251 items. Consequently, 39 articles that were identical between the two bases were found and eliminated, leaving a total of 212 articles.

#### 1.2.3. Phase 3: Eligibility

Detailed information of each article was exported from both the databases and saved in the eligibility phase. Using the Mendeley Desktop software, each article was downloaded and examined. Article-related information such as the titles, researchers' names, journal names, and years of publication were cross-checked so then each piece of information was undoubted. The detailed information of each study was stored using the Microsoft 365 Excel software. Using this software, the study's abstract, country, purpose, activities, findings, and implications were extracted and documented. This method made it simpler to organize the findings according to the context of the study, alphabet, years, and country. In addition, the tabulated summary of the findings and charts was able to be easily incorporated into the text of this article. In conclusion, for the eligibility phase, 70 out of the initial 212 papers were ineligible for synthesis because they lacked empirical data.

#### 1.2.4. Phase 4: Included

**Figure 1:**
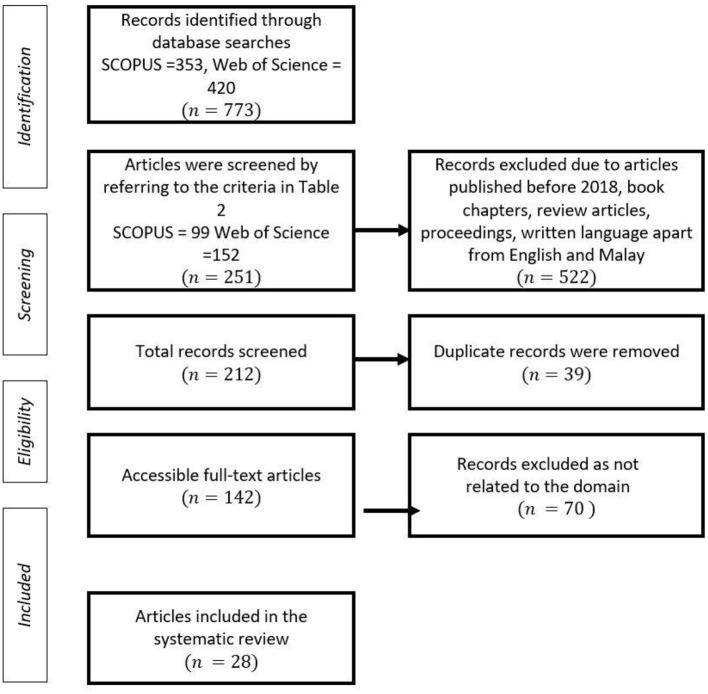
PRISMA systematics review adapted from Page et al. (2021).

The initial search highlighted 23 document results from the Scopus database and 41 document results from the Web of Science (WOS) database. There was more research conducted on the 54 qualifying papers. Ultimately, 28 documents were chosen and properly examined. Figure 1 depicts a summary of the literature review's selection procedure.

## 2. Results

A total of 28 papers were chosen through a search and selection procedure based on the goal of this study and the stated study criteria. Table 3 shows all articles selected based on the author's name, year of publication, country, and article title.

**Table 3:** Twenty eight selected articles.

**Author's name**	**Year**	**Country**	**Method**	**Article title**
Al Khateeb (2019)	2019	Jordan	Quantitative	Effect of mobile gaming on mathematical achievement among 4th graders
Alkhede and Holmqvist (2021)	2021	Sweden	Qualitative	Preschool Children's Learning Opportunities Using Natural Numbers in Number Row Activities
Altanis et al. (2018)	2018	Greece	Mixed	Systematic design and rapid development of motion-based touchless games for enhancing students' thinking skills
Baek and and Touati (2020)	2020	Korea	Quantitative	Comparing Collaborative and Cooperative Gameplay for Academic and Gaming Achievements
Barros et al. (2020)	2020	Portuguese	Qualitative	The effect of the serious game Tempoly on learning arithmetic polynomial operations
Delport (2019)	2019	South Africa	Quantitative	Numeracy students' perspectives on a new digital learning tool at a South African university
Deng et al. (2020)	2020	China	Qualitative	Digital game-based learning in a Shanghai primary-school mathematics class: A case study
Hulse et al. (2019)	2019	United State	Quantitative	From here to there! Elementary: a game-based approach to developing number sense and early algebraic understanding
Ilhan (2021)	2021	Turkey	Quantitative	The Impact of Game-Based, Modeling, and Collaborative Learning Methods on the Achievements, Motivations, and Visual Mathematical Literacy Perceptions
Juric et al. (2021)	2021	Croatia	Quantitative	Motivational Elements in Computer Games for Learning Mathematics
Kärki et al. (2021)	2021	Finland	Quantitative	Improving rational number knowledge using the NanoRoboMath digital game
Ke (2019)	2019	United State	Mixed	Mathematical problem solving and learning in an architecture-themed epistemic game
Kiili et al. (2018)	2018	Germany	Quantitative	Evaluating the effectiveness of a game-based rational number training - In-game metrics as learning indicators
Liu et al. (2021)	2021	Taiwan	Quantitative	An Integrated View of Information Feedback, Game Quality, and Autonomous Motivation for Evaluating Game-Based Learning Effectiveness
Malliakas et al. (2021)	2021	Greece	Quantitative	Educational Intervention through a Board Game for the Teaching of Mathematics to Dyslexic Greek Students
Marín-Díaz et al. (2020)	2020	Spain	Quantitative	Digital escape room, using Genial.Ly and a breakout to learn algebra at secondary education level in Spain
Moon and Ke (2020)	2020	United State	Mixed	Exploring the Relationships Among Middle School Students' Peer Interactions, Task Efficiency, and Learning Engagement in Game-Based Learning
Ramani et al. (2020)	2021	United state	Quantitative	Racing dragons and remembering aliens: Benefits of playing number and working memory games on kindergartners' numerical knowledge
Rocha and Dondio (2021)	2019	Ireland	Quantitative	Effects of a videogame in math performance and anxiety in primary school
Scalise et al. (2020)	2020	United State	Quantitative	Benefits of Playing Numerical Card Games on Head Start Children's Mathematical Skills
Suryani et al. (2019)	2019	Indonesia	Quantitative	Pengaruh model pembelajaran teams games touraments dengan permainan monopoli terhadap hasil belajar matematik di SMK kolese tiara bangsa
Tazouti et al. (2019)	2019	Marocco	Quantitative	JeuTICE: An arabic serious game to enhance mathematics skills of young children
Thai et al. (2022)	2022	United State	Quantitative	Accelerating Early Math Learning with Research-Based Personalized Learning Games: A Cluster Randomized Controlled Trial
Ting et al. (2019)	2019	Hong Kong	Quantitative	Active learning via problem-based collaborative games in a large mathematics university course in Hong Kong
van Putten et al. (2020)	2020	South Africa	Mixed	The developmental influence of collaborative games in the Grade 6 mathematics classroom
Vanbecelaere et al. (2020)	2020	Australia	Quantitative	The effects of two digital educational games on cognitive and non-cognitive math and reading outcomes
Yeh et al. (2019)	2019	Taiwan	Quantitative	Enhancing achievement and interest in mathematics learning through Math-Island
Zabala-Vargas et al. (2021)	2021	Spain	Quantitative	Strengthening Motivation in the Mathematical Engineering Teaching Processes – A Proposal from Gamification and Game-Based Learning

### 2.1. Research background

The background analysis is based on 28 papers and includes the country, year of publication, type of approach, and the respondents' education level for each.

#### 2.1.1. Countries

**Figure 2:**
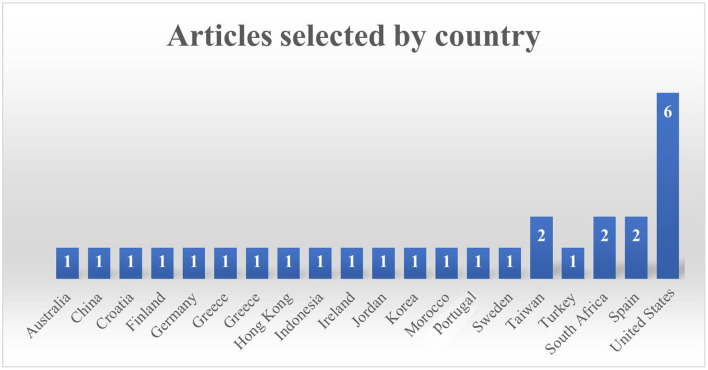
Number of articles based on country.

Twenty nations were involved in 28 articles. Six United States articles (Hulse et al., 2019; Ke, 2019; Moon and Ke, 2020; Ramani et al., 2020; Scalise et al., 2020; Thai et al., 2022), two articles from Spain (Jiménez et al., 2020; Zabala-Vargas et al., 2021), two articles from Greece (Altanis et al., 2018; Malliakas et al., 2021), two articles from South Africa (Delport, 2019; van Putten et al., 2020), and two articles from Taiwan (Yeh et al., 2019; Liu et al., 2021) were included. There was one article each from Australia (Vanbecelaere et al., 2020), China (Deng et al., 2020), Croatia (Juric et al., 2021), Finland (Kärki et al., 2021), Germany (Kiili et al., 2018), Hong Kong (Ting et al., 2019), Indonesia (Suryani et al., 2019), Ireland (Rocha and Dondio, 2021), Jordan (Al Khateeb, 2019; Baek and and Touati, 2020), Morocco (Tazouti et al., 2019), Portugal (Barros et al., 2020), Sweden (Alkhede and Holmqvist, 2021), and Turkey (Ilhan, 2021). Figure 2 shows the list of selected articles based on country.

#### 2.1.2. Year of publication

**Figure 3:**
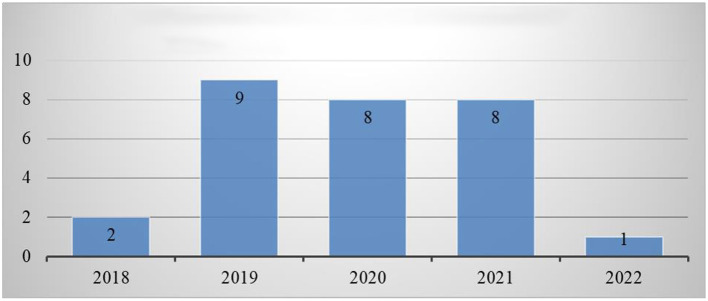
Number of articles based on year of publication.

In terms of publishing year, two articles were published in 2018 (Altanis et al., 2018; Kiili et al., 2018). In 2019, nine articles were published (Al Khateeb, 2019; Delport, 2019; Hulse et al., 2019; Ke, 2019; Suryani et al., 2019; Tazouti et al., 2019; Ting et al., 2019; Yeh et al., 2019; Rocha and Dondio, 2021). Subsequently, there were eight publications in 2020 (Baek and and Touati, 2020; Barros et al., 2020; Deng et al., 2020; Jiménez et al., 2020; Moon and Ke, 2020; Scalise et al., 2020; Vanbecelaere et al., 2020; Juric et al., 2021). Furthermore, there were eight articles (Alkhede and Holmqvist, 2021; Ilhan, 2021; Juric et al., 2021; Kärki et al., 2021; Liu et al., 2021; López et al., 2021; Malliakas et al., 2021; Zabala-Vargas et al., 2021) published in 2021. In 2022, there was only one article published (Thai et al., 2022). In summary, the majority of papers published in 2019 were chosen, followed by those published in 2020, 2021, 2018, and 2022. The list of selected articles based on year of publication is shown in Figure 3.

#### 2.1.3. Research method

**Figure 4:**
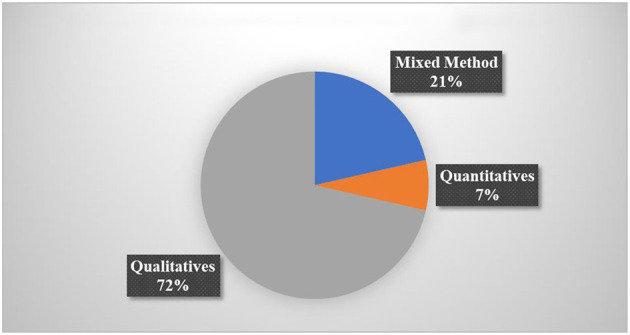
The number of articles based on the research method used.

There were three sorts of research method used: qualitative, quantitative, and mixed. As shown in Figure 4, there were 20 quantitative studies where 72% specifically used a quantitative approach (Kiili et al., 2018; Al Khateeb, 2019; Delport, 2019; Hulse et al., 2019; Suryani et al., 2019; Tazouti et al., 2019; Ting et al., 2019; Baek and and Touati, 2020; Barros et al., 2020; Jiménez et al., 2020; Ramani et al., 2020; Scalise et al., 2020; Vanbecelaere et al., 2020; Ilhan, 2021; Juric et al., 2021; Kärki et al., 2021; Liu et al., 2021; Malliakas et al., 2021; Zabala-Vargas et al., 2021; Thai et al., 2022). Six studies (21%) employed a mixed methods approach (Altanis et al., 2018; Ke, 2019; Yeh et al., 2019; Moon and Ke, 2020; Juric et al., 2021; Rocha and Dondio, 2021). Only two studies (7%) used a qualitative approach (Deng et al., 2020; Alkhede and Holmqvist, 2021).

#### 2.1.4. Respondents

**Figure 5:**
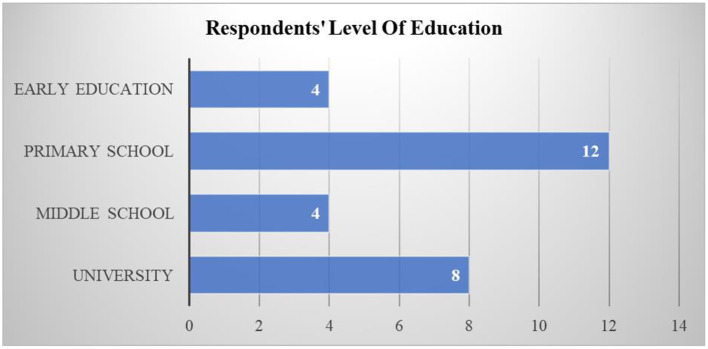
The number of articles based on the respondent's level of education.

Students served as the respondents in the selected research studies. They were categorized as being involved in early education (Kindergarten and Preschool), primary school, secondary school (Middle School and High School), and university. Four studies involved early education students (Ramani et al., 2020; Scalise et al., 2020; Alkhede and Holmqvist, 2021; Thai et al., 2022). There were 12 studies involving lower school students (Kiili et al., 2018; Al Khateeb, 2019; Hulse et al., 2019; Tazouti et al., 2019; Yeh et al., 2019; Baek and and Touati, 2020; Deng et al., 2020; Vanbecelaere et al., 2020; Ilhan, 2021; Juric et al., 2021; Kärki et al., 2021; Rocha and Dondio, 2021). Eight studies involved high school students (Altanis et al., 2018; Ke, 2019; Suryani et al., 2019; Barros et al., 2020; Jiménez et al., 2020; Moon and Ke, 2020; Juric et al., 2021; Malliakas et al., 2021). Only four studies involved university students (Delport, 2019; Ting et al., 2019; Liu et al., 2021; Zabala-Vargas et al., 2021). Figure 5 shows the number of articles based on the respondents' education level.

### 2.2. Contextual factors

The contextual factors consist of the mathematical topics examined, the types of game employed (digital or non-digital), and the programs or gaming instruments employed.

#### 2.2.1. Mathematics topics studied

**Figure 6:**
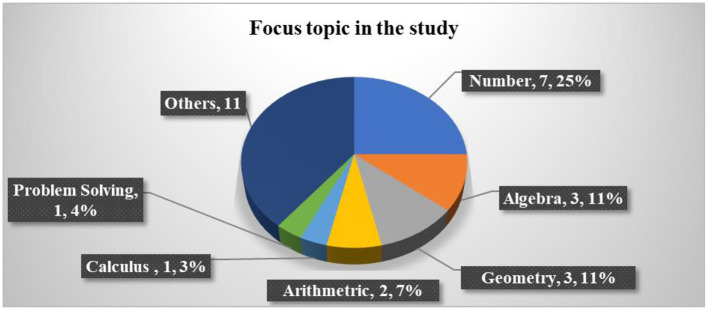
The number of articles based on the topic studied.

Several topics have been extensively blended with game-based learning (seven studies). For example, natural numbers (Alkhede and Holmqvist, 2021), rational numbers (Kiili et al., 2018; Kärki et al., 2021), numeracy (Delport, 2019), numerical skills (Vanbecelaere et al., 2020), and numerical and function skills (Ramani et al., 2020; Scalise et al., 2020). Three studies focused on geometry topics (Altanis et al., 2018; Moon and Ke, 2020; Ilhan, 2021). Three studies focused on algebra topics (Hulse et al., 2019; Barros et al., 2020; Marín-Díaz et al., 2020). Not only that but some studies focused on the topics involved in arithmetic (Yeh et al., 2019; Deng et al., 2020), calculus (Ting et al., 2019), and problem-solving (Ke, 2019). Ten studies focused only on mathematical knowledge and did not focus on specific topics (Al Khateeb, 2019; Suryani et al., 2019; Tazouti et al., 2019; Baek and and Touati, 2020; van Putten et al., 2020; Juric et al., 2021; Liu et al., 2021; Malliakas et al., 2021; Rocha and Dondio, 2021; Zabala-Vargas et al., 2021; Thai et al., 2022). Figure 6 shows the number of articles based on the topics studied.

#### 2.2.2. Utilized game type (digital and non-digital)

**Figure 7:**
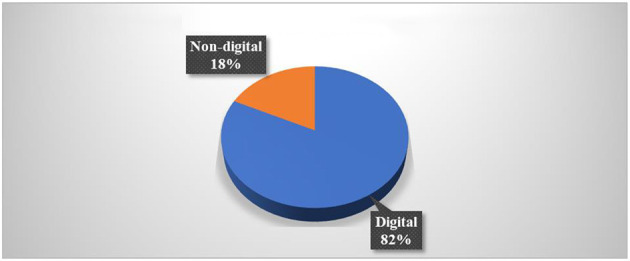
The number of articles based on the type of game used.

There were 23 studies (82%) using digital game-based learning (Altanis et al., 2018; Kiili et al., 2018; Al Khateeb, 2019; Delport, 2019; Hulse et al., 2019; Ke, 2019; Tazouti et al., 2019; Ting et al., 2019; Yeh et al., 2019; Baek and and Touati, 2020; Barros et al., 2020; Deng et al., 2020; Marín-Díaz et al., 2020; Moon and Ke, 2020; Ramani et al., 2020; Vanbecelaere et al., 2020; Juric et al., 2021; Kärki et al., 2021; Rocha and Dondio, 2021; Zabala-Vargas et al., 2021; Thai et al., 2022). Only five studies (18%) employed non-digital games (Suryani et al., 2019; Scalise et al., 2020; Alkhede and Holmqvist, 2021; Juric et al., 2021; Malliakas et al., 2021). Figure 7 shows the number of articles by type of game used.

#### 2.2.3. Utilized application or educational game tool

There were 15 studies involving games with specific names, i.e., Serious Game Tempoly (Barros et al., 2020), Minecraft (Baek and and Touati, 2020), From Here to There (FH2T:E) (Hulse et al., 2019), Wuzzit Trouble (Deng et al., 2020), Mind Tap (Delport, 2019), Team Games Tournaments (TGT) with Monopoly (Suryani et al., 2019), My Math Academy (Thai et al., 2022), JEUTICE (Tazouti et al., 2019), NanoRoboMath (Kärki et al., 2021), Zagnonetke Mudrog Lisca (Juric et al., 2021), Kinect Game (Altanis et al., 2018), Digital Escape Room (Marín-Díaz et al., 2020), Once Upon a Maths (2D Game) (Rocha and Dondio, 2021), Math-Island System (Yeh et al., 2019), E- Rebuild (Moon and Ke, 2020), and an architecture-themed epistemic game (Ke, 2019). There were five studies stating games based on topics, namely rational number training in-game metrics (Kiili et al., 2018), number row activities (Alkhede and Holmqvist, 2021), geometry instruction activities (Ilhan, 2021), a Number Sense Game (NSG) and a Reading Game (RG) (Vanbecelaere et al., 2020), and numerical card games (Scalise et al., 2020). There were four studies looking into the type of games used, namely board games (Malliakas et al., 2021), digital games (Zabala-Vargas et al., 2021), mobile games (Al Khateeb, 2019), and tablet-based training games (Ramani et al., 2020). There were two studies stating games based on learning approaches, namely collaborative games (Ting et al., 2019; Juric et al., 2021). Table 4 shows the game applications and tools used in the previous studies.

**Table 4:** Analysis of the previous studies regarding the application or game tools used.

**No**.	**Education games**	**References**	**Remarks**
1.	Serious game tempoly	Barros et al., 2020	Educational games with specific names
2.	Minecraft	Baek and and Touati, 2020	
3.	Form here to there (FH2T:E)	Hulse et al., 2019	
4.	Wuzzit trouble	Deng et al., 2020	
5.	My math academy	Thai et al., 2022	
6.	JEUTICE	Tazouti et al., 2019	
7.	NanoRoboMath	Kärki et al., 2021	
8.	Team games touraments (TGT) with monopoli	Suryani et al., 2019	
9.	Once upon a maths (2D game)	Rocha and Dondio, 2021	
10.	Math-island system	Yeh et al., 2019	
11.	Digital escape room	Marín-Díaz et al., 2020	
12.	Zagnonetke mudrog lisca	Juric et al., 2021	
13.	Kinect game	Altanis et al., 2018	
14.	Architecture-themed epistemic game	Ke, 2019	
15.	E-Rebuild	Moon and Ke, 2020	
16.	Rational number training in-game metrics	Kiili et al., 2018	Games based on mathematical topics
17.	Number sense game (NSG), reading game (RG)	Vanbecelaere et al., 2020	
18.	Number row activities	Alkhede and Holmqvist, 2021	
19.	Gometry instruction activitis	Ilhan, 2021	
20.	Numerical card games	Scalise et al., 2020	
21.	Mobile game	Al Khateeb, 2019	Games based on the type of game tools used
22.	Tablet-based training games	Ramani et al., 2020	
23.	Board game	Malliakas et al., 2021	
24.	Digital game	Zabala-Vargas et al., 2021	
25.	Collaborative games	van Putten et al., 2020	Game-based learning approach
26.	Collaborative game	Ting et al., 2019	

### 2.3. The developed theme

Learning is everywhere. Students learn mental skills, develop their attitudes, and acquire new physical skills as they perform the activities of daily living. Learning can be divided into three categories: cognitive, affective, and psychomotor. There are multiple levels of learning within each domain ranging from more basic, surface-level learning to more complex, deeper-level learning. The developed themes include the cognitive domain and affective domain. The cognitive taxonomy was described in 1956, and the affective taxonomy was described in 1964.

#### 2.3.1. Cognitive domain

The cognitive domain aims to develop the mental skills and acquisition of knowledge of the individual. Mathematical performance implies a series of numerical and mathematical skills as well as certain general cognitive abilities that, if inadequate, can have a cascading effect on mathematical learning. Twenty-eight of the chosen research studies demonstrate that game-based learning generates favorable responses in the cognitive domains. The cognitive domains manifest in 27 studies, including knowledge, skills, and the students' achievement in mathematics. The cognitive domain relates to knowledge and intellectual skills such as understanding, organizing ideas, analyzing and synthesizing information, applying knowledge, choosing among alternatives in problem solving, and evaluating ideas or actions.

There were 20 studies involving knowledge, skills, and achievement (Altanis et al., 2018; Kiili et al., 2018; Al Khateeb, 2019; Delport, 2019; Hulse et al., 2019; Ke, 2019; Suryani et al., 2019; Tazouti et al., 2019; Ting et al., 2019; Yeh et al., 2019; Baek and and Touati, 2020; Barros et al., 2020; Jiménez et al., 2020; Ramani et al., 2020; Scalise et al., 2020; Vanbecelaere et al., 2020; Alkhede and Holmqvist, 2021; Juric et al., 2021; Kärki et al., 2021; Thai et al., 2022). There are numerous cognitive functions involved in learning mathematics. Processing speed can help with simple tasks like decoding numbers and counting quickly which can help with speeding up mathematical operations. When developing training, it is critical to consider the cognitive domain and its subcategories. Activities for teaching knowledge may differ significantly from those for developing cognitive abilities such as synthesis, application, and evaluation. When a researcher considers the cognitive subcategories, they should consider developing objectives that will help the participants advance through this learning process.

#### 2.3.2. Affective domain

The affective domain is one of three domains in Bloom's taxonomy. The domain includes the manner in which people deal with things emotionally such as feeling, values, appreciation, enthusiasm, motivation, and attitudes. Gamification's main objectives are to improve certain skills, add learning objectives, engage students, optimize learning, support behavior changes, and to socialize. Twenty-eight of the selected articles have shown that game-based learning generates positive changes in the affective domains. The gamified learning approach has the potential to alter student behavior and is widely acknowledged as one of the most useful tools for creating intrinsically motivating experiences. A growing body of research demonstrates how digital games can foster and maintain high levels of learning motivation and engagement. Digital game-based learning (DGB) is thus increasingly recognized as an invaluable medium to promote emotionally engaging learning experiences. The most significant affective domain that appeared in the study is the motivation of the students when learning mathematics.

Table 5 shows the analysis of the previous studies on the cognitive and affective domains. There were a total of 20 studies found involving the students' attitude, motivation, interest or involvement (Altanis et al., 2018; Kiili et al., 2018; Al Khateeb, 2019; Delport, 2019; Hulse et al., 2019; Ke, 2019; Tazouti et al., 2019; Yeh et al., 2019; Barros et al., 2020; Deng et al., 2020; Jiménez et al., 2020; Moon and Ke, 2020; Ramani et al., 2020; Alkhede and Holmqvist, 2021; Ilhan, 2021; Juric et al., 2021; Liu et al., 2021; Rocha and Dondio, 2021; Zabala-Vargas et al., 2021; Thai et al., 2022). In detail, there are six studies related to attitude and interest, eight studies related to motivation, and six studies that examine the students' involvement in their classes.

**Table 5:** Analysis of the previous studies on the cognitive and affective domains.

**No**.	**Domain**	**Cognitive domain**			**Affective domain**			
	**Author/domian**	**Mathematical knowledge**	**Mathematical skill**	**Achievement**	**Attitude**	**Motivation**	**Interest**	**Involvement**
1	Kiili et al. (2018)	1		1	1			
2	Altanis et al. (2018)	1	1		1			
3	Ting et al. (2019)	1		1				
4	Al Khateeb (2019)			1				1
5	Yeh et al. (2019)	1		1			1	
6	Hulse et al. (2019)	1		1				1
7	Tazouti et al. (2019)	1		1		1	1	
8	Ke (2019)			1	1			1
9	Delport (2019)	1	1			1	1	1
10	Suryani et al. (2019)			1				
11	Scalise et al. (2020)		1	1				
12	Baek and and Touati (2020)			1	1			
13	Jiménez et al. (2020)	1				1		
14	Deng et al. (2020)						1	1
15	Moon and Ke (2020)							1
16	Ramani et al. (2020)	1			1			
17	van Putten et al. (2020)	1	1	1				
18	Barros et al. (2020)	1		1				1
19	Vanbecelaere et al. (2020)	1						
20	Liu et al. (2021)					1		
21	Malliakas et al. (2021)			1				
22	Rocha and Dondio (2021)			1		1		1
23	Kärki et al. (2021)	1		1				
24	Juric et al. (2021)				1	1		
25	Alkhede and Holmqvist (2021)	1					1	
26	Zabala-Vargas et al. (2021)					1		
27	Ilhan (2021)			1			1	
28	Thai et al. (2022)	1	1			1		

## 3. Discussion

### 3.1. Influence of game-based learning in mathematics education on the students' cognitive domain

According to the results of the systematic literature review, 14 of the identified studies were relevant to both the cognitive and affective domains. Five studies only focus on the affective domain, while one study focuses on the cognitive domain only. Game-based learning affects both the cognitive and affective domains. Game-based learning is a way of active teaching and learning that involves the use of commercial or educational games in the classroom. Engaging students in their learning, known as active learning, a learner-centered teaching strategy, calls for their participation in activities. To achieve a meaningful learning experience, these tasks include responding to inquiries, resolving issues, discussing the material, passing along knowledge, and externalizing cognitive processes (Yllana-Prieto et al., 2023). It is believed that identifying and implementing game elements in this process can improve learning across a variety of topics, domains, and fields of study (Alshammari, 2019). The findings of a systematic literature review show that 19 successfully identified studies are unquestionably associated with the cognitive domain. The cognitive domain involves knowledge and the development of mental or intellectual skills. There are six main categories of cognitive processes: knowledge, understanding, application, analysis, synthesis, and evaluation. The cognitive domain discusses the recollection or retention of knowledge and the development of intellectual abilities and skills. Cognitive objectives vary from readily recalling learned materials to combining and synthesizing new ideas and materials in original and creative ways. GBL helps to develop the learners' cognitive abilities, encourages problem-solving, facilitates collaboration, and raises self-esteem.

#### 3.1.1. Knowledge and skills

Games are considered to be an effective tool in education for quickening learning, teaching challenging material, and encouraging systemic thinking (Ding et al., 2018). Numbers, algebra, geometry, arithmetic, calculus, problem-solving, and mathematical topics in general are covered. Students, including those in early education, primary school, secondary school, and university, can gain mathematical knowledge and skills through game-based learning. One of the most effective learning strategies is active learning through gamification which allows the students to learn through playing games and using their classes more effectively. Training groups using video games have significantly increased their conceptual and rational amount of knowledge (Mohd et al., 2020). For example, educational digital games use software that is able to improve the students' problem solving skills and the' lessons in mathematics (Acquah and Katz, 2020). Besides this, the use of digital game-based learning in mathematics learning has been found to help students improve their memory and understanding of learning and abstract mathematical concepts. In other words, DGBL can be a bridge to connect a concrete understanding with the students' abstract understanding of mathematics. This indirectly allows them to master the concrete steps for solving various mathematical problems. Through various DGBL applications where the mathematical lesson content is adjusted to fit the game, teachers can help their students formulate situations into mathematical forms, use concepts, facts, procedures, and reasoning, and interpret, apply, and evaluate mathematical results. In addition, the exciting visuals in the DGBL application can help maintain the students' attention and working memory toward the learning activities and further help the students speed up their visual information processing through the mathematical learning activities. Not only that, in high-level thinking skills, the use of digital game-based learning, such as educational mathematics games or simulation games, also impact the students' mathematical skills, primarily through the various reasoning tasks in DGBL mathematical activities. This matter is a central element when learning mathematics as it seeks to foster logical, critical, creative, innovative, and analytical thinking to better face various mathematical problems (Mahmud et al., 2021).

#### 3.1.2. Achievement

Game-based learning has been shown to improve the students' achievements when learning. This is because this is a 21st-century learning style that stresses student-centered learning in which students learn collaboratively with their teachers and peers *via* conversations and problem-solving (Wong and Osman, 2018). The user-centered design allows the learning experiences to be psychologically accessible, not just physically. It also reflects the student's cognitive knowledge and socioemotional profile besides how their unique psychological attributes relate to the environment and pedagogical framework (Mahmud et al., 2022). However, it permits taking into account the fact that every student has unique needs that must be taken into consideration (Hernández-lara and Serradell-lopez, 2018). In addition to the incorporation of game-based learning into student education, the integration of mediated reality technology is viewed as a contributor to the students' achievements. Eighty-two percent of the research studies reviewed utilized digital games to teach mathematics. The merging of technology elements in the use of digital games as a student response system is one of the requirements that substantially stimulates the learning environment to become more engaging and significant (Bicen and Kocakoyun, 2018). This is consistent with the findings of Ding et al. (2018) who discovered that the achievement of their pupils increased when they incorporated digital games into the lesson. With the engagement of motivation, interest, attitude, and active involvement in studying mathematics through a game-based learning technique, the outcomes from the learning applications assist the students in comprehending their lesson content and ultimately enhancing their academic performance.

### 3.2. Influence of game-based learning in mathematics education on the students' affective domains

The affective domain refers to the affective reactions to a stimulus. According to research, game-based learning positively impacts on a students' affective domain in terms of attitude, motivation, involvement, interest, and confidence. In addition to the cognitive domain, appropriate tactics should take into account the affective domain such as the students' development stages, needs, abilities, talents, and interests so then the teaching and learning offered are more applicable and relevant (Ashikin and Roslinda, 2019). Besides this, mathematics learning activities through DGBL provide space for students to boost their engagement through collaborating and communicating during the learning activities which provides a good affective development space for students. There are various additional advantages of using DGBL in teaching and learning mathematics activities including socio-emotional and soft skills development. In addition, the development of the students' potential will also be increased through a positive competitive environment in DGBL-based learning activities that are conducted in a competitive manner. The healthy competition created in DGBL through progressive learning as part of the experience provides space for the students to accelerate their cognitive and emotional development, thereby increasing their self-efficacy toward learning mathematics (Mahmud et al., 2020).

#### 3.2.1. Interest and motivation

Educational games improve interest and concentration, improving the students' learning (Alonso-Fernández et al., 2020). Games also stimulate motivation because of its impact on cognitive development, affective skills, and the emotional and social states of the students (Paravizo et al., 2018). A game-based learning environment can increase the students' interest and motivation. The findings indicate that employing a game-based strategy to create an engaging, dynamic environment can help children have fun while learning. As a result, teaching and learning sessions can boost the students' interest and motivation. Students can be indirectly exposed to the idea that learning is not solely dependent on the teacher's presentation in the classroom but that it can also take place in a more engaging and effective way when they are on their own (Jasni et al., 2019). The advantages of game-based learning include accommodating the students' interests and motivation. In addition, teaching games that encourage the students to acquire greater goal orientation through increased patience, repeated learning, teamwork, and friendly rivalry during the learning process is also beneficial (Ding et al., 2018). Both variables aid the students in their attempts to comprehend and master the subjects presented, particularly mathematics. Subramaniam et al. (2022) explained that the subject of mathematics is often considered to be a difficult and boring subject. This has indirectly reduced the students' motivation and interest in learning mathematics. However, based on DGBL features that can attract the students' interest such as goals, rules, competition, challenge, fantasy, and entertainment, it is possible to provide added value and new initiatives as part of increasing the students' motivation and interest in learning mathematics (Mahmud and Law, 2022). In addition, using DGBL in mathematics learning that is carried out collaboratively has a more significant impact on the student motivation toward learning than collaborative learning activities that do not use DGBL applications. In addition, using visual rewards in the DGBL application, such as badges and tokens, also serve as a method of positive reinforcement to increase the students' interest and motivation in mathematics.

#### 3.2.2. Engagement

Engagement is a different behavioral attitude that has been found to be a useful indicator of academic performance while being positively correlated with student learning outcomes (Delfino, 2019). Students are encouraged to achieve exceptional results when learning through digital games because it is unquestionably more enjoyable. Engagement happens when someone's attention is completely focused on a specific activity. Thus, the virtual games industry indicates that engagement is a tool to keep the player's attention on the game. The method used in game-based learning is a student-centered method that requires the students' active participation throughout the lesson. This method allows the students to experience playing while learning which is more enjoyable than traditional learning methods that cause the pupils to become bored (Rebollo et al., 2022). The implementation of learning methods based on digital games can grab the students' attention, motivate them to engage themselves with learning, and raise their achievement levels (Tangkui and Keong, 2020). This game-based learning method does not require the students to be static in their respective places, only focusing on the whiteboard in front of them. This can help the teachers create a conducive and cheerful atmosphere during the teaching and learning process. In addition to being a requirement for successful educational practices, engagement can also be defined as the time and effort that students devote to their academic pursuits. There are three components to it: affection, cognition, and behavior. One teaching and learning strategy that aims to boost student engagement and make lessons more interesting is called “gamification in education” (Nisa et al., 2020). Through game-based learning activities, students can interact with digital learning materials and engage more dynamically in a fun learning environment. This is because game-based learning emphasizes the development and use of games as a tool in learning while playing and helps the teachers design lessons more interactively to help the students understand mathematical concepts in an advanced manner. This will indirectly increase the level of student engagement to help them focus on learning mathematics.

#### 3.2.3. Attitude

Game-based learning affects knowledge gains in addition to mathematical accomplishments. However, affective factors like the students' attitudes and beliefs about mathematics and its teaching are also crucial components of mathematics education because they can have a significant impact on the students' mathematical abilities and future mathematic learning (Vankúš, 2021). Attitude toward mathematics is defined as a liking or disliking of mathematics, a tendency to engage in or avoid mathematics activities, a belief that one is good or bad at mathematics and a belief that mathematics is useful or useless (Kurniasih et al., 2020). Game-based learning can make students more creative and focus better on their studies, facilitating the learning process with their friends, encouraging collaborative behavior through problem-solving, and maintaining the students' interest in the learning process (Khairuddin and Mailok, 2019). The findings of the study by Sahin and Yilmaz (2020) also proves that the use of games has a favorable and significant impact on the academic achievement and attitude of pupils. With the help of games, students gain a deeper understanding of abstract topics through 3D virtual objects with the aid of video games to achieve more meaningful learning. Based on their research, they also discovered that the students were satisfied and wished to continue using augmented reality in the future. Furthermore, game-based learning elements that emphasize competition and learning while playing makes the learning more enjoyable for the students and reduces their anxiety about math subjects. This indirectly assists the students in developing social-emotional growth and soft skills (Baul and Mahmud, 2021). Furthermore, the use of DGBL can catalyze the students' levels of self-efficacy when solving various mathematical problems through various challenging learning experience activities, as well as improving the students' social skills and communication confidence in various collaborative learning environments.

## 4. Conclusion

In conclusion, this systematic literature has analyzed 28 articles regarding the influence of game-based learning on the cognitive and affective domains of students from 2018 to 2022. Since 2019, the number of studies relating to game-based learning has increased.

In this case, the limited working memory of the students may point to a particular challenge, specifically remembering information and carrying out manipulations or operations at the same time. Thus, the use of GBL can help the students learn effectively and finish the task given in a manner that is linked to normal short-term memory. The entertainment element in GBL leads the student to learning and studying with enthusiasm. Students can even finish the task given which requires the passive repetition of elements.

Besides, the effectiveness of learning mathematics differs depending on the learning environment. As such, the use of GBL, which promotes an environment that combines game content with knowledge, will enhance the students' learning progress. The integration of the student's cognitive and affective domains with GBL can also support the learning process which creates an easier yet powerful enough environment for the students to study in.

Exploring new ways of learning through the implementation of GBL also benefits the students according to their cognitive and affective domains. Well-designed and correctly utilized GBL can improve the student's learning due to the competitive elements which encourages the students to engage in learning mathematics. The impact of the rewards act as a motivation and can also attract the student's interest in learning, especially in relation to their cognitive aspect.

However, the teachers faced difficulties when it came to inventing personal gamification tools based on the student's different mathematical knowledge in order to cater to their needs. In this case, the teachers had to allocate plenty of time to engage in the planning and designing of gamification tools. Even if this is possible, the teaching preparation and concern about the effectiveness of the learning makes the situation unwise for teacher to distribute their time according to.

The factors that influence the cognitive and affective domains of the students when they are learning are essential. Therefore, GBL should be implemented successfully in the teaching and learning of mathematics because it is said to be an excellent platform to improve learning. In short, this research proves that game-based learning is being increasingly acknowledged and incorporated in T&L. The cognitive and affective domains are used to classify diverse items. The research instruments employed, the selection of the research participants, and the game design regarding the influence of game-based learning are recommended for use in future studies.

## Data availability statement

The original contributions presented in the study are included in the article/supplementary material, further inquiries can be directed to the corresponding author.

## Author contributions

MM conceived, designed, collected the database, organized the database, and performed the analysis for the study. HH managed data collection ethics, co-wrote the manuscript, and contributed to manuscript revision. All authors read and approved the final submitted version.
